# *In vivo *microRNA-155 expression influences antigen-specific T cell-mediated immune responses generated by DNA vaccination

**DOI:** 10.1186/2045-3701-1-3

**Published:** 2011-01-18

**Authors:** Chih-Ping Mao, Liangmei He, Ya-Chea Tsai, Shiwen Peng, Tae Heung Kang, Xiaowu Pang, Archana Monie, Chien-Fu Hung, T-C Wu

**Affiliations:** 1 Department of Pathology, Johns Hopkins School of Medicine, Baltimore, Maryland, USA; 2 Department of Oral Diagnostic Service, Howard University, Washington DC, USA; 3 Department of Obstetrics and Gynecology, Johns Hopkins School of Medicine, Baltimore, Maryland, USA; 4 Department of Molecular Microbiology and Immunology, Johns Hopkins School of Medicine, Baltimore, Maryland, USA; 5 Department of Oncology, Johns Hopkins School of Medicine, Baltimore, Maryland, USA

## Abstract

**Background:**

MicroRNA (miRNA) molecules are potent mediators of post-transcriptional gene silencing that are emerging to be critical in the regulation of innate and adaptive immunity.

**Results:**

Here we report that miR-155--an oncogenic miRNA with important function in the mammalian immune system--is induced in dendritic cells (DCs) upon maturation and potentially attenuates their ability to activate T cells. Biolistic epidermal transfection with DNA encoding miR-155 suppressed the induction of antigen-specific T cell-mediated immunity, whereas reduction of endogenous miR-155 by a partially complementary antisense sequence reversed this effect. Because DCs represent a significant component of epidermal tissue and are among the most potent of antigen-presenting cells, the inhibitory actions of miR-155 could be mediated through this subset of cells.

**Conclusions:**

These results suggest that miR-155 may repress the expression of key molecules involved in lymph node migration, antigen presentation, or T cell activation in DCs, and thus forms part of a negative regulatory pathway that dampens the generation of T cell-mediated immune responses. Modulation of miR-155 expression in epidermis therefore represents a potentially promising form of gene therapy for the control of diseases ranging from autoimmunity to cancer and viral infection.

## Background

The mammalian immune system exists in a state of dynamic equilibrium, in which aggressive responses to foreign pathogens are curtailed by intrinsic feedback mechanisms that confer tolerance to self tissue. The genes that maintain this equilibrium are exquisitely regulated at the transcriptional level by intricate molecular pathways that have gradually become revealed over the past few years. However, the post-transcriptional control of these genes remains to be understood.

Recently, it was discovered that multiple aspects of cellular function are influenced by microRNA (miRNA), a diverse class of small (~22 nucleotide) and copious RNA species that efficiently 'knocks down' a vast array of genes at the post-transcriptional stage [1]. Collectively, these molecules are predicted to modulate the expression of many human genes. miRNA is initially produced as a ~60 nucleotide precursor with a defined stem-loop structure. Following nuclear export, it is processed by the enzyme Dicer into a ~22 nucleotide duplex, and a single strand is then preferentially incorporated into the RNA-induced silencing complex (RISC). The miRNA-RISC binds to partially complementary sites in the 5' or 3' untranslated regions (UTR) of target mRNA molecules, where it induces either translational repression or mRNA degradation.

Several recent reports have demonstrated that miRNAs exert profound effects on the development and function of innate and adaptive immunity (for review, see [2]). Recent studies have shown that miRNAs can control maturation, function, and maintenance of dendritic cells [3-5]. Based on these findings, we were interested in whether miRNA expression in dendritic cells (DCs), highly potent professional antigen-presenting cells, impacts their ability to activate naïve T cells. We chose to study a recently discovered miRNA, miR-155--which is encoded by exon 3 of the *bic *gene--since evidence suggests that it is intimately involved in regulation of multiple arms of the immune system [6,7]. Rodriguez *et al*. demonstrated that transgenic mice carrying an inactivating mutation in *bic *were unable to protect against challenge with *Salmonella typhimurium *despite pre-vaccination with a nonvirulent strain of these bacteria [7]. Further analysis revealed that these *bic*-deficient (*bic*^*m/m*^) mice had reduced numbers of IgM class-switched antigen-specific antibodies, and interferon (IFN)-γ and interleukin (IL)-2 secretion by activated T cells was impaired, indicating that miR-155 is broadly indispensable for normal immunological function [7]. By contrast, a recent study has reported that miR-155 attenuates the inflammatory pathway induced by IL-1 *in vitro *[8]. Thus, the function of miR-155 in the physiologic setting, and specifically whether it acts to propagate or suppress the adaptive immune response, is presently unclear. We hypothesized that--due to the pleiotropic effects of miR-155 on the immune system established in previous studies--its expression in DCs may modulate the strength of T cell priming. In this context, we sought to clarify the *in vivo *role of miR-155 in influencing DC-mediated T cell activation.

Here we show that miR-155 is induced in murine bone marrow-derived DCs (BMDCs) upon stimulation with the Toll-like receptor (TLR) 4 ligand lipopolysaccharide (LPS), and that its expression attenuates T cell activation *in vivo*. Conversely, reduction of endogenous miR-155 levels with a partially complementary antisense sequence augmented the induction of antigen-specific T cells. Our data suggest that miR-155 expression in activated DCs may be involved in the repression of genes required for antigen presentation or T cell stimulation. Thus, miR-155 may constitute part of a negative regulatory system that evolved to temper the magnitude of the adaptive immune response and protect against the onset of autoimmune pathologies.

## Results

### DCs express detectable levels of endogenous miR-155

**Figure 1:**
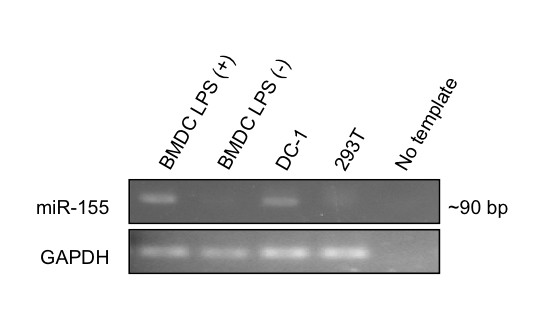
**Characterization of miR-155 expression in BMDCs using RT-PCR**. Total RNA was purified from 1 × 10^7 ^cultured BMDCs and enriched for small RNA species. Endpoint RT-PCR was then performed using primers directed against miR-155 to detect the presence of this miRNA in DCs. Expression of GAPDH was also tested to validate the assay. RNA from DC-1 and HEK 293 cells was used as a positive and negative control, respectively.

To determine whether miR-155 has biological significance in DCs, we first assayed for endogenous expression of this miRNA in BMDCs stimulated with the TLR4 agonist LPS. Endpoint RT-PCR was then performed using small RNA isolated from BMDCs with primers specific for miR-155. As shown in Figure 1, a band was observed at ~90 base pairs in the LPS-pulsed BMDC RNA-loaded lane. The location of this band is consistent with the approximate expected size of the RT-PCR-amplified miRNA product, indicating that miR-155 is present in activated DCs. Significantly, no miR-155 was observed in BMDCs left untreated with LPS, suggesting that this miRNA is induced upon maturation and thus may play an important role in the inflammatory response. The DC-1 and 293T cell lines were assessed for miR-155 expression as positive and negative controls, respectively.

### A DNA vector that encodes miR-155, *bic*^*155*^, efficiently silences expression of a reporter construct that contains an miR-155 complementary sequence

**Figure 2:**
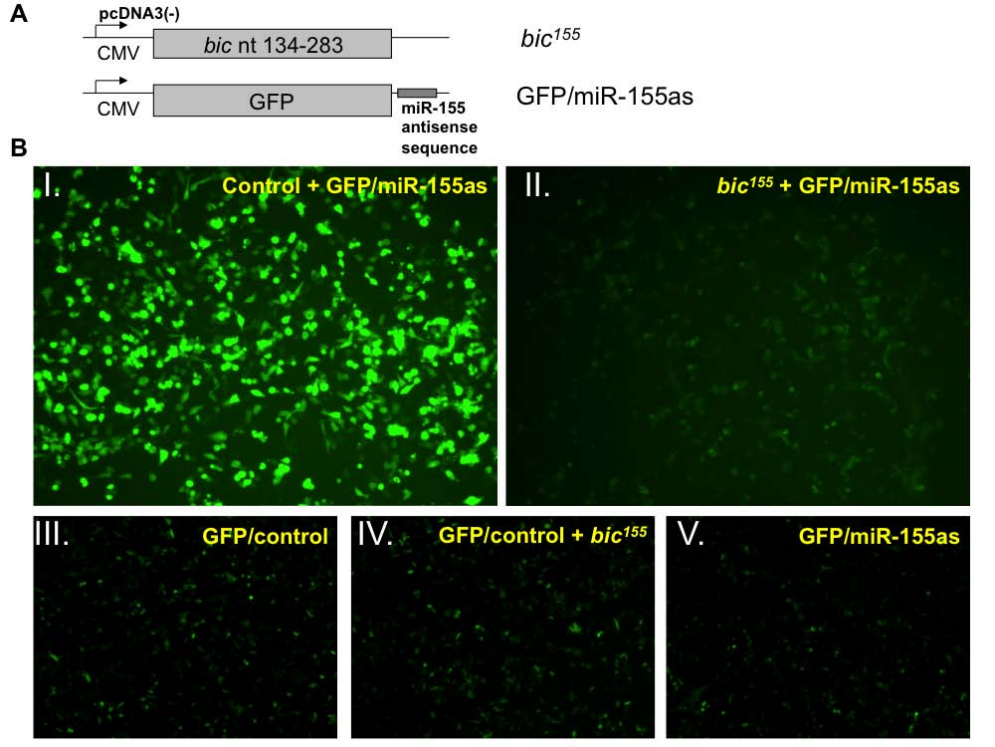
**Generation and characterization of the knockdown efficiency of *bic***^***155***^**, a mammalian expression vector encoding miR-155**. **A**, Schematic diagrams to show *bic*^*155 *^(top), a construct containing nucleotides 134-283 of exon 3 of the *bic *gene, and GFP/miR-155as (bottom), a reporter construct containing a sequence perfectly antisense to miR-155 in the 3' UTR of GFP. **B**, Fluorescence microscope image at 24 hrs of DC-1 cells transfected with GFP/miR-155as in combination with either *bic*^*155 *^(panel I) or a control construct encoding non-specific miRNA (panel II). GFP/miR-155as and *bic*^*155 *^transfection complexes were formulated in Lipofectamine 2000 in a 1:9 mass ratio. The knockdown efficiency of *bic*^*155 *^was measured as the relative difference in fluorescent intensity between the two groups. For further characterization of *bic*^*155*^, DC-1 cells were also transfected with GFP/control (panel III), GFP/control and *bic*^*155 *^(panel IV), or GFP/miR-155 (panel V).

Because miR-155 is strongly induced in DCs upon maturation, we sought to determine whether its expression holds immunological significance. In this context, we synthesized a DNA vector that encodes an active form of miR-155 in order to modulate its expression in DCs and study how it influences their ability to prime naïve antigen-specific T cells. Chung *et al*. have previously shown that the nucleotide sequence 134-283 from exon 3 of the *bic *gene is the minimal segment necessary to achieve strong expression and activity of miR-155 [9]. This sequence contains the miR-155 stem-loop flanked by portions required for efficient miRNA processing to the mature form. We generated a mammalian expression vector encoding nucleotide sequence 134-283 from exon 3 of *bic *to generate *bic*^*155 *^(Figure 2A, top). In order to characterize the knockdown capacity of *bic*^*155*^, a reporter construct was created by inserting a sequence perfectly complementary to the mature form of miR-155 into the 3' UTR of the green fluorescence protein (GFP) gene to form GFP/miR-155as (Figure 2A, bottom). We expected that cells transfected with GFP/miR-155as should display high levels of GFP expression, which would be significantly reduced by the introduction of the *bic*^*155 *^construct due to the targeted knockdown of the GFP reporter.

DC-1 cells were cotransfected with GFP/miR-155as and either *bic*^*155 *^or a control construct encoding non-specific miRNA. The cells were visualized by fluorescence microscopy 24 hrs after transfection. As shown in Figure 2B, cotransfection with *bic*^*155 *^(panel II) compared to control (panel I) strongly reduced GFP expression from the GFP/miR-155as vector. There was no noticeable difference in cellular viability or proliferation between these groups, and transfection efficiency appeared uniform as assessed by flow cytometry (data not shown). Thus, *bic*^*155 *^represents a DNA vector that efficiently produces functional miR-155 in mammalian DCs. Furthermore, as evident in panels III and IV, transfection with *bic*^*155 *^does not alter GFP expression from a reporter construct in which the miR-155 target sequence is abolished (GFP/control), confirming the specificity of our construct.

To determine the extent to which the endogenous miR-155 in DC-1 cells may have contributed to the observed knockdown effect, we compared the fluorescence intensity in cells transfected with small amounts of either GFP/control or GFP/miR-155as. It was found that GFP expression was not significantly, reduced in cells transfected with GFP/miR-155as (panel V) relative to GFP/control (panel III). Altogether these data indicate that introduction of *bic*^*155 *^in our *in vitro *system dramatically increases the expression of functional miR-155 over basal levels.

### Coadministration of CRT/E7 DNA vaccine with *bic*^*155 *^attenuates E7-specific T cell-mediated immune responses in vaccinated mice

Next we aimed to determine whether *bic*^*155 *^expression in DCs has functional significance and can influence the activation of antigen-specific T cells *in vivo*. It has been shown that epidermal DCs in mice can be efficiently transfected by intradermal DNA administration using the gene gun, a biolistic particle delivery system [10]. These transfected DCs are activated by the pressure from bombardment and undergo migration to peripheral lymphoid organs. In fact, they have been found to constitute approximately 0.2% of CD11c^+ ^cells in the inguinal lymph nodes and persist there for at least 5 days [11]. Our laboratory has previously developed a chimeric DNA vaccine encoding the molecular chaperone calreticulin linked to the E7 oncoprotein of human papillomavirus type-16 (CRT/E7) and demonstrated that vaccination with CRT/E7 can elicit potent CD8^+ ^T cell-mediated immunity against E7 [12]. We thus utilized this vaccination system to generate antigen-specific T cells *in vivo*, and investigated how the magnitude of these responses might be modulated when *bic*^*155 *^is coadministered with CRT/E7 into mice by gene gun.

**Figure 3:**
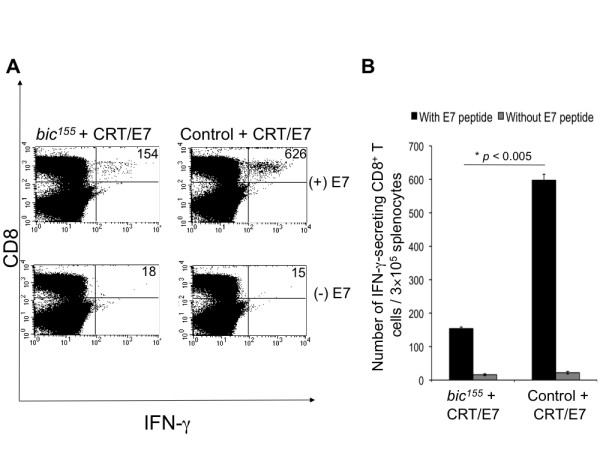
**Characterization of the number of E7-specific IFN-γ CD8^+ ^T cells in mice vaccinated intradermally with CRT/E7 DNA in combination with either *bic*^*155 *^or a control construct**. **A**, C57BL/6 mice (3 per group) were immunized with CRT/E7 DNA in combination with either *bic*^*155 *^(left panels) or a control construct (right panels). At day 7, animals received boosters at the same dose and regimen. At day 14, splenocytes were harvested, cultured for 15 hrs with (top panels) or without (bottom panels) E7 peptide, and stained for surface CD8 and intracellular IFN-γ. Cells were analyzed by flow cytometry. The top right quadrant in each dot plot indicates the number of IFN-γ-secreting E7-specific CD8^+ ^T cells. **B**, Bar graph representation of the flow cytometry data (mean ± SD).

C57BL/6 mice were intradermally administered with CRT/E7 in combination with either *bic*^*155 *^or a control construct. Animals were boosted with the same dose and regimen on day 7. Splenocytes were harvested on day 14 and cultured for 15 hrs in the presence or absence of E7 peptide. Cells were then costained for intracellular IFN-γ and surface CD8 and subsequently analyzed by flow cytometry. As demonstrated in Figure 3A, coadministration with *bic*^*155*^, compared to control, greatly decreased the number of IFN-γ-secreting, E7-specific CD8^+ ^T cells induced by CRT/E7. As expected, splenocytes in both vaccination groups not pulsed with E7 peptide contained negligible numbers of IFN-γ-secreting, E7-specific CD8^+ ^T cells. Figure 3B is a bar graph representation of the data (* *p *< 0.005). Our data suggest that miR-155 mediates an important immunosuppressive function in DCs, perhaps through the post-transcriptional knockdown of molecules that are central to T cell activation.

### A DNA vector that encodes an antisense RNA sequence partially complementary to miR-155, *I*^*155*^, rescues the expression of GFP in DCs cotransfected with *Bic*^*155 *^and GFP/miR-155as

We reasoned that, if miR-155 expression in DCs indeed negatively regulates T cell activation, then reducing endogenous levels of this miRNA should have the opposite effect. To further explore this possibility, we generated a DNA vector that encodes an antisense RNA sequence partially complementary to miR-155 and examined whether it would, as predicted, exhibit a potentiating effect on T cell activation when coadministered with CRT/E7.

**Figure 4:**
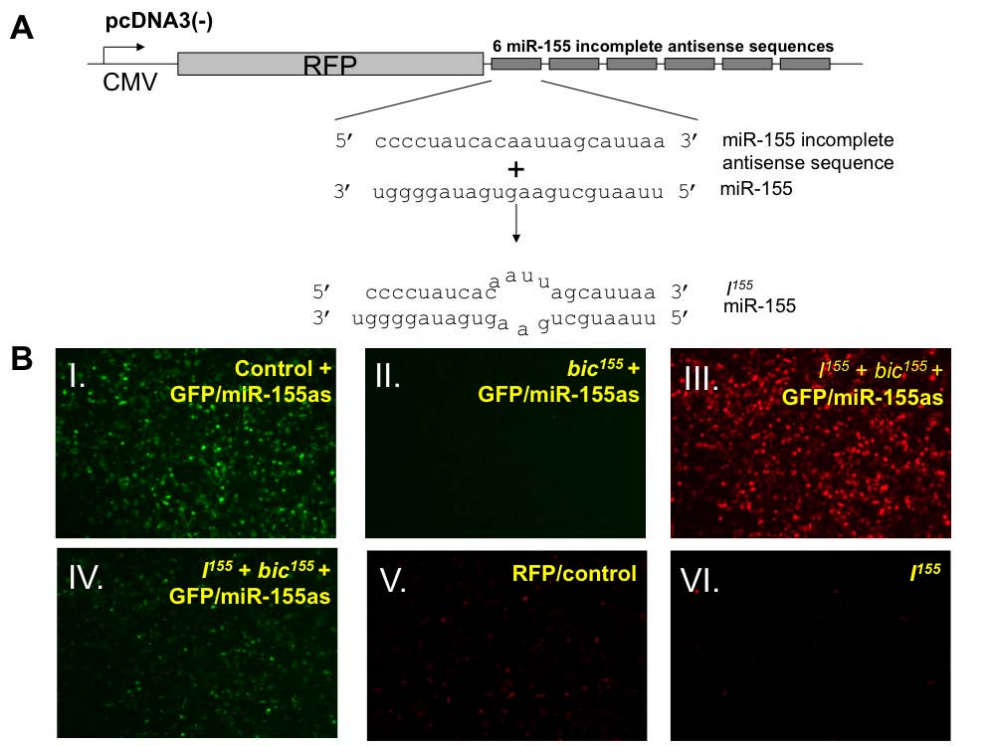
**Generation and characterization of the ability of *I***^***155***^**, a mammalian expression vector encoding sequences partially antisense to miR-155, to restore GFP expression in a DC line cotransfected with *bic***^***155***^**and GFP/miR-155as**. **A**, Schematic diagram to depict *I*^*155*^, a DNA plasmid construct that contains 6 tandemly repeated sequences partially antisense to miR-155 cloned into the 3' UTR of red fluorescent protein. The partially complementary sequences of *I*^*155 *^shown in the diagram bind imperfectly to miR-155 to form a 4-nucleotide mismatched bulged site and may serve as a competitive inhibitor of miR-155. **B**, Fluorescence microscope image at 24 hrs of DC-1 cells transfected with GFP/miR-155as in combination with a control construct encoding non-specific miRNA (panel I), *bic*^*155 *^(panel II) or both *bic*^*155 *^and *I*^*155 *^(panels III and IV). The empty pcDNA3 vector was used to standardize the total amount of transfected DNA. GFP/miR-155as and *bic*^*155 *^transfection complexes were formulated in Lipofectamine 2000 in a 1:9 mass ratio. The transfection efficiency of *I*^*155 *^was measured by the red fluorescent intensity (panel V) and the degree to which *I*^*155 *^restored GFP expression in *bic*^*155*^- and GFP/miR-155as-cotransfected cells (panel IV). To further characterize the *I*^*155 *^construct, DC-1 cells were transfected with 0.1 μg of either RFP/control (panel V) or *I*^*155 *^(panel VI).

Ebert *et al*. have recently demonstrated that partially complementary antisense RNA sequences may serve as competitive inhibitors of miRNA [13]. Based on this finding, we designed a mammalian expression vector which contains 6 tandemly repeated miR-155 partially antisense regions cloned into the 3' UTR of the red fluorescence protein (RFP) gene to generate *I*^*155*^. Figure 4A shows a schematic diagram of *I*^*155*^. The miR-155 partially antisense regions have been shown to bind stably to miR-155-RISC and form bulges at the site normally cleaved by Argonaute-2, the catalytic component of RISC, effectively protecting *I*^*155 *^from degradation by the cellular RNA interference machinery [13]. Furthermore, the presence of multiple partially antisense repeats has been shown to enhance the avidity between *I*^*155 *^and miR-155-RISC and enables this miRNA inhibitor to successfully outcompete endogenous miR-155 target genes [13]. *I*^*155 *^contains the RFP gene to facilitate verification of its transfection efficiency as well as the intracellular stability of its encoded RNA.

We sought to determine whether the expression of *I*^*155 *^can inhibit the activity of miR-155 in transfected DC-1 cells. As shown in Figure 2B, transfection of DC-1 cells with GFP/miR-155as generated a strong green fluorescent signal in these cells, which was markedly diminished by introduction of *bic*^*155*^. In theory, if expression of *I*^*155 *^leads to the knockdown of miR-155, then treatment of cells with *I*^*155 *^should rescue GFP expression in cells cotransfected with GFP/miR-155as and *bic*^*155*^.

DC-1 cells were transfected with GFP/miR-155as alone or in combination with either *bic*^*155 *^or both *bic*^*155 *^and *I*^*155*^. Cells were imaged by fluorescence microscopy 24 hrs after transfection. As illustrated in Figure 4B, green fluorescence was observed in cells cotransfected with GFP/miR-155as and a control construct encoding non-specific miRNA (panel I), whereas it was virtually undetectable in cells also cotransfected with GFP/miR-155as and *bic*^*155 *^(panel II), confirming previous observations that *bic*^*155 *^exerts a dramatic knockdown effect on miR-155 target gene expression (see Figure 2B). Further, we observed that transfection of DC-1 cells with *I*^*155 *^together with GFP/miR-155as and *bic*^*155 *^restored the expression of GFP (panel IV). The transfection efficiency of *I*^*155 *^in DC-1 cells was demonstrated by the expression of RFP (panel III). There was no noticeable difference in cellular viability or proliferation between all these groups, and transfection efficiency appeared uniform as assessed by flow cytometry (data not shown). Therefore, our data suggest that *I*^*155 *^acts as a highly efficient inhibitor of miR-155.

Because the *I*^*155 *^construct contains 6 tandemly repeated sites partially complementary to miR-155, it is conceivable that--in addition to its role as an inhibitor--it could also serve as a target for endogenous miR-155, and its expression could be suppressed at the post-transcriptional level. To test this possibility, we compared red fluorescence intensity in DCs transfected with a small amount of either *I*^*155 *^or an RFP vector with the miR-155 antisense sequences abolished (RFP/control). As shown in Figure 4B, cells transfected with *I*^*155 *^(panel VI), relative to RFP/control (panel V), displayed substantially decreased RFP expression, although this difference in expression could be due to variation in the nature of the DNA constructs. There was no observable difference in confluence or transfection efficiency between the groups. Thus, we have demonstrated that *I*^*155 *^potently attenuates the expression and function of miR-155 in DCs and itself serves as a target of the endogenous form of this miRNA.

### Coadministration of *I*^*155 *^with CRT/E7 DNA enhances E7-specific CD8^+ ^T cell-mediated immune responses in vaccinated mice

**Figure 5:**
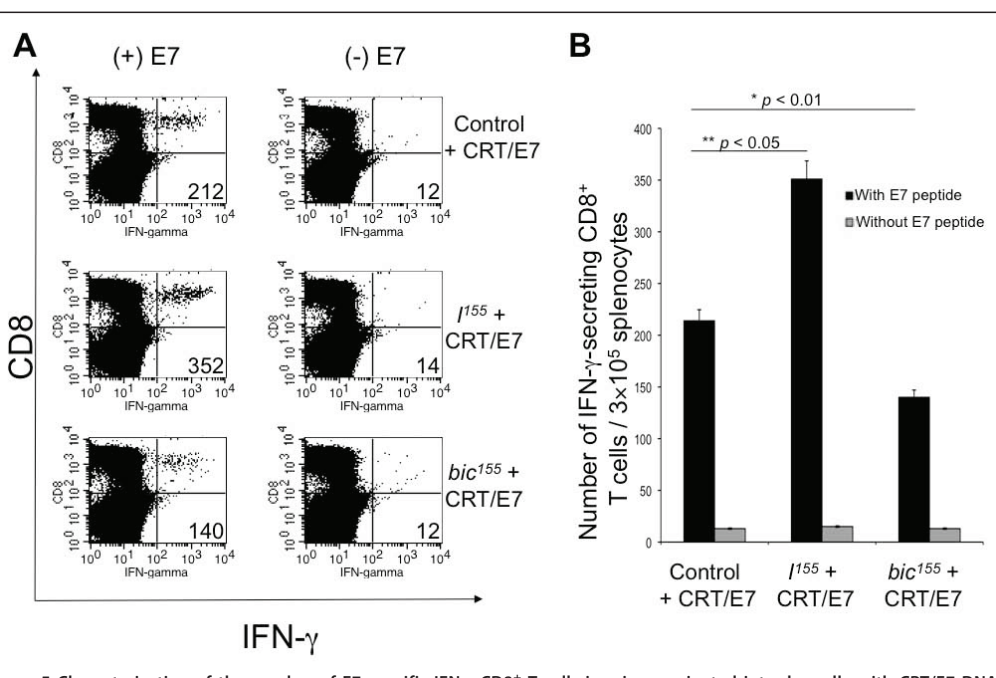
**Characterization of the number of E7-specific IFN-γ CD8^+ ^T cells in mice vaccinated intradermally with CRT/E7 DNA in combination with *bic*^*155 *^, *I*^*155*^, or a control construct**. C57BL/6 mice (3 per group) were immunized with CRT/E7 in combination with *bic*^*155*^, *I*^*155*^, or a control construct. At day 7, animals received boosters at the same dose and regimen. At day 14, splenocytes were harvested, cultured for 15 hrs with (black bars) or without (grey bars) E7 peptide, and stained for surface CD8 and intracellular IFN-γ. Cells were analyzed by flow cytometry. **A**, Representative flow cytometry data depicting the number of E7-specific CD8^+ ^T cells. **B**, Bar graph representation of the flow cytometry data (mean ± SD). The *y*-axis on the bar graph depicts the number of IFN-γ-secreting E7-specific CD8^+ ^T cells per 3 × 10^5 ^counted splenocytes (mean ± SD).

We hypothesized that, since *bic*^*155 *^strongly attenuated the T cell-mediated immune response in CRT/E7-vaccinated mice, an inhibitor of miR-155, *I*^*155*^, would reverse this effect. C57BL/6 mice were intradermally administered by gene gun with CRT/E7 in combination with *bic*^*155*^, *I*^*155*^, or a control construct. Animals were boosted with the same dose and regimen on day 7. Splenocytes were harvested on day 14 and then characterized for E7-specific CD8^+ ^T cell-mediated immune responses by intracellular IFN-γ and surface CD8 staining followed by flow cytometry analysis. As shown in Figure 5, while coadministration of *bic*^*155 *^with CRT/E7 DNA decreased the number of IFN-γ-secreting E7-specific cytotoxic T cells generated by CRT/E7 (* *p *< 0.01), coadministration of *I*^*155 *^with CRT/E7 DNA significantly improved the E7-specific CD8^+ ^T cell-mediated immune response generated by CRT/E7 (** *p *< 0.05). Altogether, our data indicate that modulation of expression levels of miR-155 can influence the ability of DCs to prime antigen-specific naïve T cells *in vivo*. Furthermore, these results suggest that miR-155 may act broadly as a post-transcriptional silencer in negative feedback pathways that dampen the adaptive immune response following the initial phases of T cell activation.

## Discussions

In the current study, we have provided data to demonstrate that miR-155 is induced in DCs upon maturation and that its expression *in vivo *negatively regulates the induction of antigen-specific T cells. Administration of *bic*^*155*^, a miR-155 expression vector, to epidermal DCs *in vivo *suppressed the generation of T cell-mediated immunity. This outcome was reversed by decreasing endogenous miR-155 levels in DCs with a partially antisense inhibitor, *I*^*155*^. These results altogether suggest that miR-155 has an inhibitory effect on DC-mediated T cell activation. In addition, our data also suggest that miRNA levels in DCs may be manipulated to modulate the activation of antigen-specific T cell-mediated immunity for therapeutic purposes.

Our findings reveal a new way in which miRNAs may exert control over the immune system. The role of miRNAs as key regulators of the innate and adaptive immune systems is becoming increasingly evident. Indeed, miRNAs have recently been implicated in a wide variety of immunological processes, including hematopoietic cell differentiation[14], lymphocyte development and function [6,7,15-17], as well as protective responses against bacterial [18] or viral [19] pathogens. The current study identifies a novel role of miRNA in the regulation of DC-mediated antigen-specific T cell priming.

In general, the expression of miRNAs is precisely controlled at the transcriptional level, enabling them to finely adjust cellular behavior as necessary. miR-155 production is believed to be transiently under the control of nuclear factor (NF)-κB and can be strongly induced during inflammation [18,20,21]. Recently, miR-155 has also been shown to play a role in the promotion of T cell-dependent tissue inflammation [22]. It has been shown that basal miR-155 levels in murine macrophages are virtually undetectable but increase dramatically by 6 hrs after treatment with the TLR ligands polyriboinosinic:polyribocytidylic acid (poly(I:C)), LPS, CpG, and P3C, as well as the cytokines IFN-β and IFN-γ, in a c-Jun N-terminal kinase-dependent manner [18,20]. We have demonstrated that this phenomenon is conserved in DCs, as stimulation of these cells with LPS strongly induced miR-155. Collectively, our findings suggest that miR-155 expression may be a global consequence of inflammation, although the precise role it plays in this process may vary depending on cell type.

It has been demonstrated that miR-155 is critical for the generation of robust T cell-mediated immunity in mice, partly through the action of this miRNA in DCs [7]. In this context, we were surprised to find that coadministration of DNA encoding miR-155 with CRT/E7 led to a decrease in E7-specific CD8^+ ^T cell-mediated immune responses *in vivo*. These results suggested to us that miR-155 may have a complicated function in DCs, in which its influence on T cell stimulatory capacity could vary depending on its expression level or the developmental stage of the cell. Alternatively, because our biolistic delivery method transfects a wide variety of epidermal cells in addition to DCs, it is conceivable that the phenomenon we observed is due to the effects of miR-155 on these other types of cells. We reasoned that if overexpression of miR-155 in DCs diminished antigen-specific T cell responses, then suppression of this miRNA should correspondingly augment these responses. Biolistic delivery to epidermal DCs of an efficient miR-155 repressor, *I*^*155*^, significantly amplified the number of IFN-γ-secreting E7-specific CD8^+ ^T cells generated by the CRT/E7 vaccine. Therefore, manipulation of endogenous miR-155 levels in DCs alters the intensity of DC-induced T cell-mediated immunity *in vivo *in a systematic manner, consistent with the proposed immunosuppressive activity of this miRNA.

Several reported studies may support the observed function of miR-155 as an attenuator of the adaptive immune system and provide clues about the molecular mechanisms through which this miRNA exerts its effects. It was recently shown that the NF-κB signaling pathway may be repressed by miR-155 through the silencing of inhibitor of NF-κB (IκB) kinase (IKK) ε [23]. In response to TLR signaling, the IKK family of proteins phosphorylates IκB, thereby enabling NF-κB to translocate into the nucleus and initiate transcription. IKKε has also been demonstrated to facilitate NF-κB nuclear import by phosphorylating its c-Rel subunit [24]. In addition, IKKβ as well as a variety of other central immunological molecules--such as Fas-associated death domain protein (FADD) and receptor (TNFRSF)-interacting serine-threonine kinase 1 (Ripk1)--likely represent major direct targets of miR-155 [21]. FADD is an adaptor molecule that is crucial for the development of normal immune responses [25,26], and Ripk1 mediates tumor necrosis factor-induced activation of NF-κB [27]. Therefore, since the maturation and T cell stimulatory capacity of DCs depends vitally on signaling through the NF-κB pathway, miR-155 could exert its inhibitory effects by suppressing this pathway on multiple levels. Furthermore, a recent study has reported that miR-155 produced by human monocyte-derived DCs attenuates the TLR/IL-1 pathway and silences transforming growth factor-β activated kinase 1 binding protein (TAB2), an important intermediate in the IL-1 cascade [8]. This *in vitro *evidence directly supports our results which suggest that miR-155 expression in DCs is part of a negative feedback loop that suppresses the inflammatory response. While we believe, on the basis of its likely molecular targets, that miR-155 influences DC biology primarily at the level of antigen presentation and processing or costimulation, we cannot exclude the possibility that the *in vivo *effects we observed in this study were at least partially due to other factors, such as the potential ability of miR-155 to impair cellular migration to the peripheral lymphoid organs. These alternative mechanisms present an important area for future investigation.

On the basis of our current data and the work of others, it is intriguing to speculate about the purpose of miR-155 activity in DCs. We propose a dose-dependent paradigm in which a threshold miR-155 expression level must be maintained in DCs in order to generate effective T cell-mediated immunity against foreign pathogens. However, when environmental stimuli cause the concentration of cellular miR-155 to escalate beyond this basal level, miR-155 becomes sufficiently abundant to repress the translation of molecules involved in antigen presentation or T cell activation, and thus may form part of a negative regulatory pathway that restrains the magnitude of the adaptive immune response and perhaps evolved to protect against the onset of autoimmune pathologies.

The current study mainly focuses on an immature DC line, DC-1. It will be important to also consider performing experiments to interfere with miR-155 levels during different maturation stages of monocyte-derived DCs (moDCs) for a more comprehensive understanding of the effect of miR-155 on the biological function of moDCs [28]. However, the potential limitations for such an experimental approach is the overexpression of miRNAs to non-physiological levels that may lead to altered target genes. Thus it is important to consider the expression levels of miRNAs in moDCs for gain- or loss-of-function studies to illustrate the influence of miR-155 on moDCs.

Finally, this study provides an impetus and framework for the development of miRNA-based therapeutics. To our knowledge, this is the first report of *in vivo *miRNA delivery for the modulation of adaptive immunity. We have demonstrated the general principle that miRNAs or their inhibitors can be biolistically administered into epidermal DCs to systematically manipulate T cell-mediated immune responses. It is exciting to envision the possibility that, in the near future, these small yet powerful RNA species may be incorporated into medicines for autoimmune disorders or into vaccines for cancers and viral infections.

## Conclusions

Taken together, our results suggest that miR-155 may repress the expression of key molecules involved in lymph node migration, antigen presentation, or T cell activation in DCs, and thus forms part of a negative regulatory pathway that dampens the generation of T cell-mediated immune responses. Modulation of miR-155 expression in DCs therefore represents a potentially promising form of gene therapy for the control of diseases ranging from autoimmunity to cancer and viral infections.

## Methods

### Preparation of BMDCs

Bone marrow cells were flushed from tibiae and femurs of C57BL/6 mice. After lysis of erythrocytes, cells were washed twice and resuspended at 1 × 10^6 ^cells/ml in RPMI-1640 medium supplemented with 2 mM glutamine, 1 mM sodium pyruvate, 100 μM nonessential amino acids, 5 × 10^-5 ^M β-mercaptoethanol, 100 IU/ml penicillin, 100 μg/ml streptomycin, 5% fetal bovine serum, and 20 ng/ml recombinant murine GM-CSF (PeproTech, Rock Hill, NJ). Cells were cultured in 24-well plates at 37°C in a humidified incubator with 5% CO_2_. On days 2 and 4, cells were replenished with fresh medium supplemented with 20 ng/ml GM-CSF. On day 6, cells were either pulsed with LPS for 5 hrs or left untreated and then harvested for RNA isolation and RT-PCR.

### miRNA isolation and detection

Total RNA was purified from 1 × 10^7 ^BMDCs by acid-phenol:chloroform extraction using the *mir*Vana miRNA Isolation Kit (Ambion, Austin, TX). The resultant sample was enriched for small RNA species by ethanol precipitation according to the manufacturer's instructions. Endpoint RT-PCR was then performed with primers directed against either mature miR-155 or the housekeeping gene GAPDH using the *mir*Vana qRT-PCR miRNA Detection Kit (Ambion). In order to facilitate the PCR, the miR-155 RT primer was designed to introduce a flanking sequence which extends beyond the 5' region of the mature form of this miRNA. Therefore, the final amplified product is about 90 base pairs. As a positive control, we used DC-1 cells, which are derived from an immortalized DC line [29] kindly provided by Dr. Kenneth Rock (University of Massachusetts, Worcester, MA) and constitutively produce miR-155. As a negative control, we used the human embryonic kidney cell line 293. Tissue from kidney has previously been shown to lack miR-155 [6].

### Plasmid expression vectors

For the generation of *bic*^*155*^, nucleotides 134-283 from exon 3 of the *bic *gene were synthesized (GeneArt, Regensburg, Germany) with flanking XbaI and EcoRI restriction sites and cloned into the pcDNA3.1(-) mammalian expression vector (Invitrogen, Carlsbad, CA). GFP/miR-155as was created by ligating an oligonucleotide (5'-CCCCTATCACAATTAGCATTAA-3') perfectly antisense to mature miR-155 into the BamHI/HindIII sites of the pcDNA3.GFP plasmid [30]. A miRNA control DNA construct was generated in which a non-specific miRNA was cloned into the pcDNA3 plasmid. This control construct is not expected to hybridize with miR-155 target sites. GFP/control represents the GFP/miR-155as construct with the miR-155 binding site deleted. For the production of *I*^*155*^, 6 tandemly repeated DNA fragments of imperfect complementarity (5'-CCCCTATCACGAAAGCATTAA-3') to mature miR-155 were synthesized with 4 guanine nucleotide spacers in between each one. These sequences were then inserted into the 3' UTR (at BamHI/HindIII sites) of pcDNA3.RFP. RFP/control represents the *I*^***155***^**construct with the miR-155 antisense region deleted**. The design of the CRT/E7 construct has been reported previously [12]. Plasmids were confirmed by DNA sequencing.

### Cell culture and DNA transfection

By continuous passage we generated a subclone (DC-1) of an immortalized DC line,[29] that is readily transfectable with Lipofectamine 2000 (Invitrogen). DC-1 cells were cultured in RPMI-1640 (Sigma-Aldrich, St. Louis, MO) at 37°C in a humidified incubator with 5% CO_2_. 5 × 10^5 ^cells/well were seeded in 6-well plates, and transfections were performed using Lipofectamine 2000 (Invitrogen) with combinations of GFP/miR-155as, *bic*^*155*^, and *I*^*155 *^in a 1:9:10 mass ratio. Empty pcDNA3 vector was added to standardize the total amount of DNA in each group to 10 μg. 24 hrs after transfection, cells were visualized by fluorescence microscopy.

### Mice

6- to 8-week old female C57BL/6 mice were purchased from the National Cancer Institute (Frederick, MD) and housed in the animal facility at CRBII, Johns Hopkins School of Medicine (Baltimore, MD). All procedures were performed in accordance with established protocols and recommendations for the proper use and care of laboratory animals.

### Biolistic DNA delivery *in vivo*

DNA-coated gold particles were prepared and delivered by a helium-driven gene gun (BioRad, Hercules, CA) using a method published previously.[31] 0.2 μg of CRT/E7 in combination with 1.8 μg of *bic*^*155*^, *I*^*155*^, or a control construct were administered to the shaved abdominal region of C57BL/6 mice (3 per group) at a discharge pressure of 400 psi. Mice were boosted at day 7 with the same dose and regimen. At day 14, the animals were sacrificed, and their spleens were explanted for further analysis.

### Flow cytometry

Splenocytes harvested from DNA-bombarded mice were pooled and seeded at 1 × 10^7 ^cells/well in 24-well plates. Cells were incubated for 15 hrs in the presence of GolgiStop (Pharmingen, San Diego, CA) with or without 1 μg/ml E7 peptide (amino acids 49-57). Cells were then washed in FACScan buffer, and surface marker detection of CD8 and intracellular cytokine staining for IFN-γ were performed as described [31]. Samples were analyzed on a Becton Dickinson FACSan Flow Cytometer with CellQuest software (BD Biosciences, San Jose, CA).

### Statistical Analysis

Data presented as mean ± standard deviation are representative of at least two different experiments. All *p*-values < 0.05 were considered significant. Statistical analysis was performed using T-test for independent samples with SigmaPlot (Systat software, Inc., San Jose, CA).

## List of abbreviations

(BMDCs): *bic*-deficient (*bic*^*m/m*^), bone marrow-derived DCs; (CRT): calreticulin; (DCs): Dendritic cells; (FADD): Fas-associated death domain protein; (GFP): green fluorescence protein; (IκB): inhibitor of NF-κB; (IKK): inhibitor of NF-κB kinase; (IFN): interferon; (IL): interleukin; (LPS): lipopolysaccharide; (miRNA): MicroRNA; (poly(I:C)): polyriboinosinic:polyribocytidylic acid; (Ripk1): receptor-interacting serine-threonine kinase 1; (RFP): red fluorescence protein; (RISC): RNA-induced silencing complex; (TLR): Toll-like receptor; (UTR): untranslated regions;

## Competing interests

The authors declare that they have no competing interests.

## Authors' contributions

CPM conceived of the study and participated in its design, execution and writing of the manuscript. LH and YCT generated the constructs and performed some of the experiments included in the figures of the manuscript. SP and THK were involved in acquisition of data. XWP was involved in the planning of the experiments. AM assisted in data interpretation and drafting the manuscript. CFH and TCW were involved in conceiving the study and were responsible for overseeing the project. All authors read and approved the final manuscript.

## References

[B1] Ambros V microRNAs: tiny regulators with great potential Cell 2001 107 823 826 10.1016/S0092-8674(01)00616-X 11779458

[B2] Lodish HF Zhou B Liu G Chen CZ Micromanagement of the immune system by microRNAs Nat Rev Immunol 2008 8 120 130 10.1038/nri2252 18204468

[B3] Liu X Zhan Z Xu L Ma F Li D Guo Z Li N Cao X MicroRNA-148/152 impair innate response and antigen presentation of TLR-triggered dendritic cells by targeting CaMKIIalpha J Immunol 2010 185 7244 7251 10.4049/jimmunol.1001573 21068402

[B4] Kuipers H Schnorfeil FM Brocker T Differentially expressed microRNAs regulate plasmacytoid vs. conventional dendritic cell development Mol Immunol 2010 48 333 340 10.1016/j.molimm.2010.07.007 20822813

[B5] Kuipers H Schnorfeil FM Fehling HJ Bartels H Brocker T Dicer-dependent microRNAs control maturation, function, and maintenance of Langerhans cells in vivo J Immunol 2010 185 400 409 10.4049/jimmunol.0903912 20530258

[B6] Thai TH Calado DP Casola S Ansel KM Xiao C Xue Y Murphy A Frendewey D Valenzuela D Kutok JL Regulation of the germinal center response by microRNA-155 Science 2007 316 604 608 10.1126/science.1141229 17463289

[B7] Rodriguez A Vigorito E Clare S Warren MV Couttet P Soond DR van Dongen S Grocock RJ Das PP Miska EA Requirement of bic/microRNA-155 for normal immune function Science 2007 316 608 611 10.1126/science.1139253 17463290 PMC2610435

[B8] Ceppi M Pereira PM Dunand-Sauthier I Barras E Reith W Santos MA Pierre P MicroRNA-155 modulates the interleukin-1 signaling pathway in activated human monocyte-derived dendritic cells Proc Natl Acad Sci USA 2009 106 2735 2740 10.1073/pnas.0811073106 19193853 PMC2650335

[B9] Chung KH Hart CC Al-Bassam S Avery A Taylor J Patel PD Vojtek AB Turner DL Polycistronic RNA polymerase II expression vectors for RNA interference based on BIC/miR-155 Nucleic Acids Res 2006 34 e53 10.1093/nar/gkl143 16614444 PMC1435982

[B10] Huang B Mao CP Peng S Hung CF Wu TC RNA interference-mediated in vivo silencing of fas ligand as a strategy for the enhancement of DNA vaccine potency Hum Gene Ther 2008 19 763 773 10.1089/hum.2007.059 18627219 PMC2587268

[B11] Kim TW Hung CF Ling M Juang J He L Hardwick JM Kumar S Wu TC Enhancing DNA vaccine potency by coadministration of DNA encoding antiapoptotic proteins J Clin Invest 2003 112 109 117 12840065 10.1172/JCI17293 PMC162284

[B12] Cheng WF Hung CF Chai CY Hsu KF He L Ling M Wu TC Tumor-specific immunity and antiangiogenesis generated by a DNA vaccine encoding calreticulin linked to a tumor antigen J Clin Invest 2001 108 669 678 11544272 10.1172/JCI12346 PMC209378

[B13] Ebert MS Neilson JR Sharp PA MicroRNA sponges: competitive inhibitors of small RNAs in mammalian cells Nat Methods 2007 4 721 726 10.1038/nmeth1079 17694064 PMC3857099

[B14] Chen CZ Li L Lodish HF Bartel DP MicroRNAs modulate hematopoietic lineage differentiation Science 2004 303 83 86 10.1126/science.1091903 14657504

[B15] Li QJ Chau J Ebert PJ Sylvester G Min H Liu G Braich R Manoharan M Soutschek J Skare P miR-181a is an intrinsic modulator of T cell sensitivity and selection Cell 2007 129 147 161 10.1016/j.cell.2007.03.008 17382377

[B16] Zhou B Wang S Mayr C Bartel DP Lodish HF miR-150, a microRNA expressed in mature B and T cells, blocks early B cell development when expressed prematurely Proc Natl Acad Sci USA 2007 104 7080 7085 10.1073/pnas.0702409104 17438277 PMC1855395

[B17] Xiao C Calado DP Galler G Thai TH Patterson HC Wang J Rajewsky N Bender TP Rajewsky K MiR-150 controls B cell differentiation by targeting the transcription factor c-Myb Cell 2007 131 146 159 10.1016/j.cell.2007.07.021 17923094

[B18] Taganov KD Boldin MP Chang KJ Baltimore D NF-kappaB-dependent induction of microRNA miR-146, an inhibitor targeted to signaling proteins of innate immune responses Proc Natl Acad Sci USA 2006 103 12481 12486 10.1073/pnas.0605298103 16885212 PMC1567904

[B19] Lecellier CH Dunoyer P Arar K Lehmann-Che J Eyquem S Himber C Saib A Voinnet O A cellular microRNA mediates antiviral defense in human cells Science 2005 308 557 560 10.1126/science.1108784 15845854

[B20] O'Connell RM Taganov KD Boldin MP Cheng G Baltimore D MicroRNA-155 is induced during the macrophage inflammatory response Proc Natl Acad Sci USA 2007 104 1604 1609 17242365 10.1073/pnas.0610731104 PMC1780072

[B21] Tili E Michaille JJ Cimino A Costinean S Dumitru CD Adair B Fabbri M Alder H Liu CG Calin GA Croce CM Modulation of miR-155 and miR-125b levels following lipopolysaccharide/TNF-alpha stimulation and their possible roles in regulating the response to endotoxin shock J Immunol 2007 179 5082 5089 17911593 10.4049/jimmunol.179.8.5082

[B22] O'Connell RM Kahn D Gibson WS Round JL Scholz RL Chaudhuri AA Kahn ME Rao DS Baltimore D MicroRNA-155 promotes autoimmune inflammation by enhancing inflammatory T cell development Immunity 2010 33 607 619 20888269 10.1016/j.immuni.2010.09.009 PMC2966521

[B23] Lu F Weidmer A Liu CG Volinia S Croce CM Lieberman PM Epstein-Barr virus-induced miR-155 attenuates NF-kappaB signaling and stabilizes latent virus persistence J Virol 2008 82 10436 10443 10.1128/JVI.00752-08 18753206 PMC2573162

[B24] Harris J Oliere S Sharma S Sun Q Lin R Hiscott J Grandvaux N Nuclear accumulation of cRel following C-terminal phosphorylation by TBK1/IKK epsilon J Immunol 2006 177 2527 2535 16888014 10.4049/jimmunol.177.4.2527

[B25] Kabra NH Kang C Hsing LC Zhang J Winoto A T cell-specific FADD-deficient mice: FADD is required for early T cell development Proc Natl Acad Sci USA 2001 98 6307 6312 10.1073/pnas.111158698 11353862 PMC33464

[B26] Zhang Y Rosenberg S Wang H Imtiyaz HZ Hou YJ Zhang J Conditional Fas-associated death domain protein (FADD): GFP knockout mice reveal FADD is dispensable in thymic development but essential in peripheral T cell homeostasis J Immunol 2005 175 3033 3044 16116191 10.4049/jimmunol.175.5.3033 PMC3110086

[B27] Ting AT Pimentel-Muinos FX Seed B RIP mediates tumor necrosis factor receptor 1 activation of NF-kappaB but not Fas/APO-1-initiated apoptosis EMBO J 1996 15 6189 6196 8947041 PMC452440

[B28] Jurkin J Schichl YM Koeffel R Bauer T Richter S Konradi S Gesslbauer B Strobl H miR-146a is differentially expressed by myeloid dendritic cell subsets and desensitizes cells to TLR2-dependent activation J Immunol 2010 184 4955 4965 10.4049/jimmunol.0903021 20375304

[B29] Shen Z Reznikoff G Dranoff G Rock KL Cloned dendritic cells can present exogenous antigens on both MHC class I and class II molecules J Immunol 1997 158 2723 2730 9058806

[B30] Hung CF Cheng WF Hsu KF Chai CY He L Ling M Wu TC Cancer immunotherapy using a DNA vaccine encoding the translocation domain of a bacterial toxin linked to a tumor antigen Cancer Res 2001 61 3698 3703 11325841

[B31] Chen CH Wang TL Hung CF Yang Y Young RA Pardoll DM Wu TC Enhancement of DNA vaccine potency by linkage of antigen gene to an HSP70 gene Cancer Res 2000 60 1035 1042 10706121

